# Motivational profiles and change in physical activity during a weight loss intervention: a secondary data analysis

**DOI:** 10.1186/s12966-021-01225-5

**Published:** 2021-12-04

**Authors:** Danielle M. Ostendorf, Sarah J. Schmiege, David E. Conroy, Suzanne Phelan, Angela D. Bryan, Victoria A. Catenacci

**Affiliations:** 1 Division of Endocrinology, Metabolism, and Diabetes, Department of Medicine, University of Colorado Anschutz Medical Campus, Campus Box C263, 12348 E. Montview Boulevard, Aurora, CO 80045 USA; 2 Department of Biostatistics and Informatics, Colorado School of Public Health, University of Colorado Anschutz Medical Campus, Aurora, CO USA; 3 Department of Kinesiology and Human Development and Family Studies, The Pennsylvania State University, University Park, State College, PA USA; 4 Department of Preventive Medicine, Northwestern University, Chicago, IL USA; 5 Department of Kinesiology, California Polytechnic State University, San Luis Obispo, CA USA; 6 Department of Psychology and Neuroscience, University of Colorado Boulder, Boulder, CO USA

**Keywords:** Motivation, Exercise, Weight loss, Obesity, Latent profile analysis

## Abstract

**Background:**

High levels of moderate-to-vigorous intensity physical activity (MVPA) are strongly associated with sustained weight loss, however the majority of adults are unsuccessful in maintaining high levels of MVPA long-term. Our goal was to identify profiles based on exercise motives, and examine the association between motivational profile and longitudinal changes in MVPA during a weight loss intervention.

**Methods:**

Adults with overweight or obesity (*n* = 169, mean ± SE; age 39 ± 0.7 years, BMI 34.4 ± 0.3 kg/m^2^, 83% female) underwent an 18-month behavioral weight loss program, including 6 months of supervised exercise, followed by 6 months of unsupervised exercise. Participants self-reported behavioral regulations for exercise at baseline (BREQ-2). Latent profile analysis identified subgroups from external, introjected, identified, and intrinsic regulations measured at baseline. Mean differences in device-measured total MVPA were compared across motivational profiles at baseline, after 6 months of supervised exercise and after a subsequent 6 months of unsupervised exercise.

**Results:**

Three motivational profiles emerged: high autonomous (high identified and intrinsic, low external regulations; *n* = 52), high combined (high scores on all exercise regulations; *n* = 25), and moderate combined (moderate scores on all exercise regulations; *n* = 92). Motivational profile was not associated with baseline level of MVPA or the increase in MVPA over the 6-month supervised exercise intervention (high autonomous: 21 ± 6 min/d; high combined: 20 ± 9 min/d; moderate combined: 33 ± 5 min/d; overall *P* > 0.05). However, during the transition from supervised to unsupervised exercise, MVPA decreased, on average, within all three profiles, but the high autonomous profile demonstrated the least attenuation in MVPA (− 3 ± 6 min/d) compared to the moderate combined profile (− 20 ± 5 min/d; *P* = 0.043).

**Conclusions:**

Results were in alignment with the Self-Determination Theory. Adults motivated by autonomous reasons (value benefits of exercise, intrinsic enjoyment) may be more likely to sustain increases in MVPA once support is removed, whereas participants with moderate-to-high scores on all types of exercise regulations may need additional long-term support in order to sustain initial increases in MVPA.

**Clinical trial registration:**

NCT01985568. Registered 24 October 2013.

**Supplementary Information:**

The online version contains supplementary material available at 10.1186/s12966-021-01225-5.

## Background

High levels of moderate-to-vigorous physical activity (MVPA) are strongly associated with sustained weight loss [1, 2], and current guidelines recommend high levels of PA for weight management [3]. However, behavioral weight loss interventions have been relatively unsuccessful in producing sustained changes in physical activity (PA) in both clinical and research settings [4, 5] and limited data exist on factors that are associated with long-term adherence to PA [6]. Given that the majority of individuals initiating a new behavior fail at sustaining that behavior over time [7], it is important to identify baseline factors that influence PA adherence within a behavioral weight loss program in order facilitate the design of more effective, tailored weight loss interventions [8, 9].

One critical factor in long-term adherence to PA within a weight loss program may be an individual’s initial motivation for exercise [10]. Self-Determination Theory (SDT) identifies different types of behavioral regulations that underlie a behavioral goal [11]. These regulations span a continuum of self-regulation that is anchored by intrinsic regulation (the most autonomous form of motivation) and external regulation (the most controlled form of motivation). Autonomous motives reflect behaviors that are more self-determined, such as exercising because the benefits of the activity are strongly valued (identified regulation) or the activity is rewarding on its own (intrinsic regulation); whereas controlled motives reflect behaviors that are less self-determined, such as exercising to attain an external reward or avoid an external punishment (external regulation) or to avoid guilt (introjected regulation) [12].

Previous studies have identified a beneficial role of autonomous motives with respect to PA adherence [10, 13–16]. However, these studies have often utilized traditional, variable-centered approaches (e.g. correlation, regression), which may obscure important individual level differences in motivation for PA. In addition, motivational regulations may not be mutually exclusive [17]. Person-centered approaches, in contrast to variable centered approaches, may improve our understanding of motivation for PA because they allow us to identify subgroups of people based on their similarities on a set of variables (i.e., different motivation regulations) [18]. Latent profile analysis is a person-centered approach that allows us to understand how different exercise regulations co-exist within an individual [19]. More recently, cross-sectional studies have used a person-centered analysis to identify motivational profiles based on exercise regulations in adults [19–25]. These studies identified 3–6 motivational profiles, including a “self-determined” or “autonomous” profile (high scores on autonomous, low scores on controlled motives), “moderate” profile (moderate scores on all regulations), “high combined” or “motivated” profile (high scores on all regulations), “low motivation” (moderate scores on external regulation and low scores on all other regulations), “controlled motivation” or “non self-determined” (high scores on external and introjected regulations and low-moderate scores on identified and intrinsic regulations), and “high introjected” or “moderate introjected” (moderate or high introjected regulation, low external regulation, and low-moderate levels of identified and intrinsic regulation). Of these cross-sectional studies, four [19, 20, 25, 26] examined whether PA was different across motivational profiles (three self-reported PA; one device-measured PA). Results suggested that adults with an autonomous or a high combined profile self-reported the highest levels of PA [19, 20, 25, 26] compared to other profiles. Although these studies lay a foundation for using a person-centered approach to understand motivation for PA, they are limited by their cross-sectional design, and/or use of self-reported PA, which can suffer from inaccuracies and bias [27]. Further, no previous study has examined how longitudinal changes in device-measured MVPA differ across motivational profiles identified at baseline in the context of a behavioral weight loss intervention.

Data from a recently completed, comprehensive behavioral weight loss intervention provided a unique opportunity to examine motivational profiles for exercise at baseline and their association with changes in device-measured MVPA over 12 months in adults with overweight or obesity [28]. Given the critical role of PA in maintaining weight loss [1], identification of whether PA adherence is different between motivational profiles in the context of a behavioral weight loss program may allow us to develop more effective weight loss interventions. It is possible that each of the different motivational profiles might benefit from unique intervention approaches, representing a more targeted intervention to improve long-term adherence to PA.

The aims of this study were to conduct a secondary analysis of existing data to 1) identify baseline motivational profiles in adults with overweight or obesity enrolled in a behavioral weight loss intervention, and 2) examine cross-sectional and longitudinal associations between profile membership and device-measured MVPA. It was hypothesized that approximately 3–4 distinct motivational profiles would form from exercise regulations measured at baseline, representing similar profile groups found in prior literature. It was also hypothesized, based on SDT, that motivational profile would be associated with changes in MVPA over time, such that participants with a motivational profile characterized by high scores on autonomous motives and low scores on controlled motives (i.e., a high autonomous profile) would demonstrate the greatest increases in MVPA in response to a behavioral weight loss program as compared to participants with less self-determined motivational profiles.

## Methods

### Participants

This secondary analysis utilized data from 169 participants (83.4% female) who participated in a randomized clinical trial designed to evaluate the optimal time to initiate exercise within a behavioral weight loss intervention and provided complete data on motivation for exercise at baseline [28]. The study was conducted at the University of Colorado Anschutz Medical Campus and approved by the Colorado Multiple Institutional Review Board (ClinicalTrials.gov NCT01985568). The methods and main study results have been previously published [28]. In brief, men and women age 18–55 years, body mass index (BMI) 27–42 kg/m^2^, who lived or worked within 20 miles of the University of Colorado Anschutz Health and Wellness Center (CU-AHWC) were recruited through campus emails and flyers, and advertisements in local newspapers. Exclusionary criteria for the parent trial included history of diabetes mellitus, cardiovascular disease, uncontrolled hypertension, or uncontrolled thyroid disease; cancer within the past 5 years (except skin cancer); any physical or medical condition that contraindicates exercise; previous bariatric surgery; eating disorder; current use of medications known to affect appetite, weight or energy metabolism; current alcohol or substance abuse; regular nicotine use; weight change of > 5% over the past 6 months; or current engagement in high levels of exercise (self-report of ≥ 150 min/week at moderate intensity or greater). Pregnant or lactating women were also excluded.

### Randomization

**Fig. 1:**
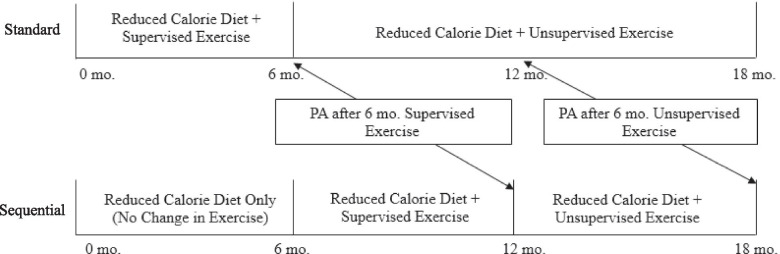
Aligned Assessment Period by Randomized Group. To ensure equal exposure to PA in analyses, PA was captured during two time points: 1) after 6 months of supervised exercise (month 6 for standard, month 12 for sequential), and 2) after 6 months of subsequent unsupervised exercise (month 12 for standard, month 18 for sequential); PA: physical activity

Participants in the parent trial were randomized to one of two groups: standard behavioral therapy (standard) or sequential behavioral therapy (sequential) in a 1:1 ratio, stratified by sex. Both standard and sequential groups received identical 18-month group-based weight loss programs and identical 6-month supervised exercise interventions. Standard participants received the supervised exercise program and exercise behavioral support during months 0–6. Sequential participants were asked not to begin exercise during months 0–6, and received a supervised exercise program and exercise behavioral support during months 7–12. In this secondary analysis, both randomized groups were combined because the supervised exercise intervention was identical and there were no differences between randomized groups in change in weight or MVPA at 18 months or change in MVPA over time using an aligned dataset (see Fig. 1); however randomized group was tested as a potential covariate and/or moderator. To ensure equal exposure to the PA intervention, changes in MVPA were analyzed using the aligned dataset (i.e. after 6 months of supervised exercise (month 6 for standard; month 12 for sequential) and after a subsequent 6 months of unsupervised exercise (month 12 for standard; month 18 for sequential; Fig. 1).

### Diet and exercise interventions

The comprehensive behavioral weight loss program in the parent trial was designed to meet current obesity treatment guidelines [29]. Participants were provided an individualized weight loss calorie goal (1200–1800 kcal/day) and asked to keep weekly food logs. Group meetings, led by a registered dietitian, were separate for each randomized condition, and were held weekly during weeks 0–20, every other week during weeks 21–26, and monthly during weeks 27–78.

The 6-month supervised exercise program was designed to progress to 300 min/week of moderate-intensity aerobic exercise and followed the recommended guidelines for weight management [30, 31]. Volume of moderate-intensity exercise increased from 20 min sessions, 3 days/week to 60 min sessions, 5 days/week over 6 months. Throughout the 6-month supervised exercise program, participants in both randomized groups were asked to perform 3 exercise sessions/week at the CU-AHWC fitness center; participants were allowed to choose the type of exercise performed during each session (treadmill, elliptical, upright or recline bike, etc.). At weeks 16 and 21, participants were asked to add additional on-own exercise sessions, bringing the total number of exercise sessions to 4 (weeks 16–20) and 5 (weeks 21–26). During on-own exercise sessions, participants were allowed to use cardiovascular exercise machines, participate in group exercise classes at the CU–AHWC fitness center, or exercise outside the CU-AHWC fitness center. Exercise intensity was monitored for all sessions using heart rate monitors; on-own session performance was verified by study staff cross-referencing PA logs with heart rate monitor data.

In addition to direct supervision of exercise sessions, participants in both randomized groups were provided six, 45 min, exercise behavioral support sessions each month during the 6-month supervised exercise phase. These one-on-one exercise support sessions were delivered at the CU-AHWC fitness center by one of two trained interventionists (with exercise certifications from an organization accredited by the National Commission for Certifying Agencies) using a standardized curriculum adapted from the PA-specific content of the multicomponent, comprehensive, behavioral weight loss curriculum [28]. Sessions focused on goal setting, outcome expectations, ways to increase exercise enjoyment, overcoming barriers to exercise, introduction to strength training, and planning for continued exercise after supervised exercise ends. Of note, the exercise behavioral support sessions were not designed with the SDT explicitly in mind. Adherence to the exercise prescription was reviewed every 2 weeks with participants by study staff. Upon completion of the 6-month supervised exercise program, participants in both groups had access to the CU-AHWC fitness center for the duration for the 18 month study and were encouraged to continue to maintain 300 min/week of moderate-intensity aerobic exercise. However, exercise was no longer supervised or monitored.

### Measures

#### Physical activity assessment

The primary outcome, total MVPA, was assessed at baseline (prior to randomization), after 6 months of supervised exercise (month 6 for standard, month 12 for sequential), and after 6 months of subsequent unsupervised exercise (month 12 for standard, month 18 for sequential) with the SenseWear Mini Research Grade Armband (BodyMedia Inc., Pittsburgh PA). The SenseWear Mini is a wireless activity monitor that is worn on the upper arm and integrates motion data from a tri-axial accelerometer with several physiological sensors (heat flux, skin temperature and galvanic skin response). The data are reduced to estimate minute-by-minute energy expenditure and classified by activity level (sedentary, light, moderate, vigorous) using a proprietary recognition pattern [32]. The SenseWear Mini has high test-retest reliability [33] and validity in free-living conditions [32, 34]. Participants were asked to wear the device 24 h/day over 7 days, which achieves intra-class correlations of more than 80% in most populations and provides the opportunity to sample behavior on week and weekend days [35]. To be included in the analysis, participants must have had ≥ 4 valid days, including ≥ 1 valid weekend day (valid day: 95% wear time).

#### Motivation for exercise

Motivation for exercise was assessed at baseline with the Behavioral Regulation in Exercise Questionnaire (BREQ-2) [36], which provides separate continuous scores (range 0 [not true for me] to 4 [very true for me]) along the self-regulation continuum. The self-regulation continuum includes amotivation (no purpose for exercise); controlled motives which includes external regulation (“I exercise because others say I should”) and introjected regulation (“I feel guilty when I don’t exercise”); and autonomous motives which includes identified regulation (“I value the benefits of exercise) and intrinsic regulation (“I exercise because it is fun”) [37]. BREQ-2 responses showed high internal consistency in our sample for each exercise regulation (Cronbach α; amotivation: .82, external: .83, introjected: .79, identified: .72, intrinsic: .91). Previous studies have confirmed the BREQ-2’s reliability and factorial, convergent, discriminant, and predictive validity [38, 39]. The questionnaire was self-administered in paper form, during an in-person visit with study staff. Participants were provided a quiet space, without study staff present, to complete the questionnaire.

#### Anthropometric and demographic characteristics

At baseline, body weight (kg) was measured with a calibrated digital scale (to the nearest 0.1 kg), height was measured with a wall-mounted stadiometer (to the nearest 0.1 cm), and waist circumference in centimeters was measured at the superior iliac crest with a tape measure by trained study staff. Age, sex, race, and ethnicity were self-reported.

### Statistical analysis

Statistical analyses were performed with Mplus (version 8.0; Muthen and Muthen, 1998–2017) and SAS (University Edition, Version 9.4 of the SAS System for Microsoft, SAS Institute Inc., Cary, NC, USA). Latent profile analysis was used to identify homogenous subgroups based on an individual’s value for exercise regulations measured at baseline. This technique is used to determine whether a categorical latent variable underlies the measured variables. We included 4 indicators (external regulation, introjected regulation, identified regulation, and intrinsic regulation) measured at baseline. Models were estimated iteratively by increasing the number of subgroups until the best-fitting model was observed. Two to seven latent profile solutions were estimated during the class enumeration process to determine the optimal number of classes. Consistent with recent latent profile analysis methodological work [40], four different formulations for the covariance structure were tested. The model covariance structure that allowed indicator variance terms to vary across latent classes but constrained within-class covariances among indicators to zero was superior and is presented herein (see Additional file 1, Supplementary Table S1 for results on other covariance/variance structures empirically examined as part of the class enumeration process). In addition to evaluating class solutions for substantive relevance (theoretical coherence and interpretability of the latent classes), recommended fit statistics were used to evaluate the fit of different solutions to the data [18, 41]. Specifically, better model fit was indicated by a lower Bayesian Information Criterion (BIC) and/or Consistent Akaike Information Criteria (CAIC) values, a significant bootstrap likelihood ratio test (BLRT; indicates that the target profile solution fits better with the data than a profile solution with 1 less profile). In addition, theoretical meaningfulness and overall interpretability of the latent classes were considered when choosing the best solution. Fit statistics and overall interpretability favored three classes and the resulting three-class model was interpretable in terms of homogeneity (i.e., individuals within a given class are similar to each other with respect to item responses) and separation (i.e., individuals across two classes are dissimilar with respect to item responses) (Table 1). The three-class model had the lowest BIC and CAIC as well as significant *P* values for the adjusted Lo-Mendell-Rubin Likelihood Ratio Test and BLRT.

**Table 1:** Fit Statistics, Homogeneity, and Separation

**Fit Statistics**									
							**H0: K classes; H1: K + 1 classes**		
Model (K-class)	**Model Log-likelihood**	**Number of parameters**	**AIC**	**BIC**	**CAIC**	**AWE**	**LRT**	**Adj LMR** ***P*** **value**	**Bootstrapped LRT** ***P*** **value**
1-class	− 937.06	8	1890.12	1915.16	1923.16	1980.20	–	–	–
2-class	− 868.06	17	1770.12	1823.33	1840.33	1961.54	135.08	0.0003	< 0.0001
3-class	− 835.14	26	1722.28	1803.66	1829.66	2015.03	64.45	0.0203	< 0.0001
4-class	− 812.95	35	1695.90	1805.44	1840.44	2089.99	43.44	0.2813	0.0400
5-class	− 798.62	44	1685.95	1822.95	1866.95	2180.67	28.06	0.7423	0.3750
**Homogeneity for 3-class Model** (values > 0.90 = low degree of homogeneity, values < 0.60 = high degree of homogeneity in bold)									
	**External**	**Introjected**	**Identified**	**Intrinsic**	**Class Label**				
class 1	0.78	0.77	0.64	**0.50**	Moderate Combined				
class 2	**0.13**	1.17	**0.50**	**0.25**	High Autonomous				
class 3	**0.18**	**0.44**	**0.30**	**0.19**	High Combined				
**Separation for 3-class Model (Cohen’s d)** (values < 0.85 = low separation; values > 2.0 = high separation in bold)									
		**External**	**Introjected**		**Identified**			**Intrinsic**	
class 1 vs. 2		1.90	0.46		1.71			**2.68**	
class 1 vs. 3		1.09	1.42		**2.16**			**2.49**	
class 2 vs. 3		**5.10**	0.72		0.30			0.38	

Fit statistics from class enumeration process using latent profile analysis where covariances were fixed to zero, but variances were allowed to differ across classes; *AIC* Akaike Information Criteria, *BIC* Bayesian Information Criteria, *CAIC* Consistent Akaike’s Information Criteria, *AWE* Approximate Weight of Evidence Criterion, *LRT* Likelihood ratio test, *Adj LMR* Adjusted Lo-Mendell-Rubin Likelihood Ratio Test; Homogeneity presented as within-class variance term, with low values indicating whether individuals within a class are similar to each other with respect to item responses. Separation presented as Cohen’s d, with high values indicating that individuals across two classes are dissimilar with respect to item responses

Multinomial logistic regression was used to examine whether baseline factors were different between motivational profiles. A Wald test (which is typically used in in mixture models to test pairwise comparisons between profiles [42]) was used to compare mean differences in MVPA at baseline, after 6 months of supervised exercise and after a subsequent 6 months of unsupervised exercise across motivational profiles, taking into account classification uncertainty using the BCH weighting method [43] with the type I error rate fixed at 0.05. The BCH method avoids shifts in the latent class solution when examining the association between the profile solution and a distal outcome [43]. Change in MVPA was examined using a completer’s analysis as well as imputing missing MVPA values using a baseline observation carried forward approach. The completer’s analysis was considered primary because we were interested in identifying whether MVPA was different across motivational profile, under optimal conditions (i.e. those who completed the 18-month intervention). Dropout rates at 12 months as a function of motivational profile were also examined, using a Chi-Square test. In addition to randomized group, several potential covariates and/or moderators were considered, including age, sex, race, ethnicity, baseline BMI, and waist circumference. The role of randomized group on the association between motivational profile and changes in MVPA was tested using a linear mixed model with change in total MVPA as the dependent variable, and motivational profile, randomized group, and the interaction between motivational profile and randomized group as the independent variables. An exploratory linear regression analysis was conducted to examine whether each covariate modified the association between motivational profile and change in MVPA. Unless otherwise indicated, data are presented as mean ± standard error. No a-priori power analysis was conducted for these secondary analyses.

## Results

### Baseline characteristics

**Fig. 2:**
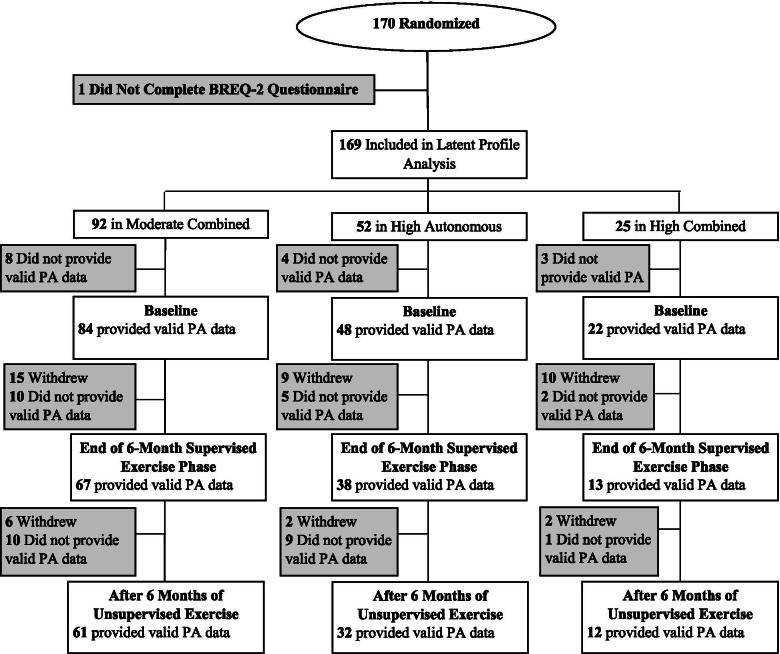
Consort Diagram. PA: physical activity

One hundred seventy participants started the intervention. One participant did not complete the BREQ-2 questionnaire at baseline, thus 169 participants were included in this analysis (Fig. 2). Participant baseline characteristics are described in Table 2. Participants with valid PA data included 154 (91%) at baseline, 118 (70%) after 6 months of supervised exercise, and 105 (62%) after a subsequent 6 months of unsupervised exercise. After 6 months of supervised exercise and after a subsequent 6 months of unsupervised exercise, participants with valid PA data compared to participants with invalid or missing PA data were not different with regards to randomized group, motivational profile, ethnicity, race, or baseline weight, BMI, or waist circumference. However, participants with valid PA data after 6 months of supervised exercise were on average older compared to those with invalid/missing data (40.9 ± 0.8 vs. 35.6 ± 1.3 years; *P* < 0.01). In addition, participants with valid PA data after 6 months of unsupervised exercise were less likely to be women (78% vs. 92% women; *P* = 0.02), more likely to be older (40.8 ± 0.9 vs. 36.9 ± 1.1 years; *P* = 0.01), and more likely to have higher levels of baseline MVPA (65.6 ± 4.4 vs. 52.9 ± 3.1; *P* = 0.02).

**Table 2:** Motivational Profile and Baseline Factors

Baseline Factors	Total Sample (*n* = 169)	Moderate Combined (*n* = 92)	High Autonomous (*n* = 52)	High Combined (*n* = 25)	Overall ***P*** value
**Randomized Group**					**0.040**
*Standard*	85 (50%)	54 (64%)	19 (22%)	12 (14%)	
*Sequential*	84 (50%)	38 (45%)	33 (39%)	13 (15%)	
**Age (y)**	39 ± 0.7	40 ± 1	39 ± 1	37 ± 2	0.46
**Anthropometric Measures**					
*BMI (kg/m2)*^*a*^	34.4 ± 0.3	34.5 ± 0.4	34.2 ± 0.6	34.8 ± 0.8	0.81
*Waist Circumference (cm)*	107.0 ± 0.8	108.2 ± 1.1	105.5 ± 1.6	105.5 ± 2.0	0.27
**Total MVPA (min/d)** ^b^	61 ± 3	60 ± 4	60 ± 6	69 ± 10	0.61
**Sex**					**0.048**
*Women*	141 (83.4%)	71 (50%)	49 (35%)	21 (15%)	
*Men*	28 (16.6%)	21 (75%)	3 (11%)	4 (14%)	
**Race**					0.80
*White*	129 (76.3%)	72 (56%)	37 (29%)	20 (16%)	
*Black*	28 (16.6%)	13 (46%)	11 (39%)	4 (14%)	
*Other*	12 (7.1%)	7 (58%)	4 (33%)	1 (8%)	
**Ethnicity**					0.59
*Hispanic or Latino*	42 (24.9%)	23 (55%)	11 (26%)	8 (19%)	
*Not Hispanic or Latino*	127 (75.2%)	69 (54%)	41 (32%)	17 (13%)	

Results (displayed as mean ± SE or n (%)); Overall *P* values reflect the overall difference across class membership and baseline factors, analyzed using multinomial logistic regression; Statistically significant *P* values (*P* < 0.05) are indicated in bold

^a^ *BMI* Body Mass Index

^b^ *n* = 84 for Moderate Combined; *n* = 48 for High Autonomous; *n* = 22 for High Combined; *MVPA* moderate-to-vigorous physical activity

### Exercise regulations

Baseline means of each exercise regulation were as follows: amotivation: 0.29 ± 0.04, external: 1.40 ± 0.09, introjected: 1.83 ± 0.08, identified: 2.67 ± 0.06, and intrinsic: 2.39 ± 0.08. The majority of participants (66%) scored 0 for amotivation regulation for exercise and the median score for the other 34% who scored above 0 was 0.75 (interquartile range 0.75). Given that participants did not use the full range of this scale and there was little-to-no variability in amotivation scores, amotivation was not used as part of the motivational profile analysis. Thirty-two percent of participants scored 0 for external regulation for exercise (skewness = 1.05, kurtosis = 0.38, Shapiro-Wilk test for normality *P* < 0.01), therefore, external regulation was recoded into 4 categories: 0 (0 ± 0; *n* = 54), 1 (> 0 but ≤ 0.5; 0.37 ± 0.02; *n* = 34), 2 (> 0.5 but ≤ 1.25; 0.93 ± 0.03; *n* = 40), and 3 (> 1.25; 2.07 ± 0.09; *n* = 41) and this recoded variable was treated as continuous during the class enumeration process.

### Identifying motivational profiles

**Fig. 3:**
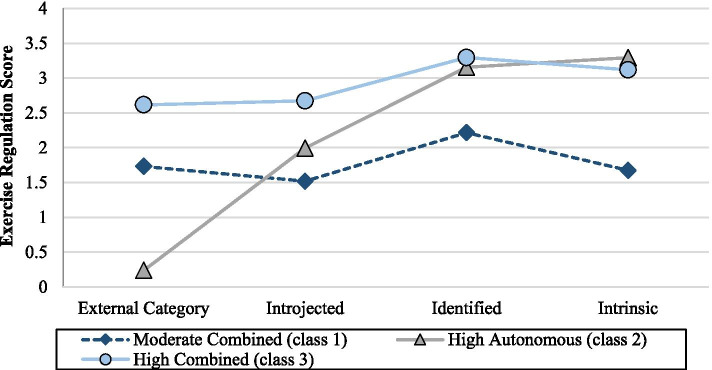
Mean Exercise Regulation Score across Baseline Motivational Profiles**.** Exercise regulation score (mean) across the four motivational profiles; Exercise regulations included external category (range 0–3; 4 categories include: 0 (score = 0), 1 (score > 0 but ≤ 0.5), 2 (score > 0.5 but ≤ 1.25), and 3 (score > 1.25), introjected (range 0–4), identified (range 0–4), and intrinsic regulations (range 0–4); *n* = 92 for Moderate Combined; *n* = 52 for High Autonomous; *n* = 25 for High Combined

Three motivational profiles were observed. The largest overall class, moderate combined (*n* = 92; 54.4%), was characterized by moderate scores on all exercise regulations (Fig. 3), and had a high degree of homogeneity for intrinsic regulation (Table 1). The second largest class, high autonomous (*n* = 52; 30.8%), was characterized by low scores on external regulation, moderate scores on introjected regulation, and high scores on identified and intrinsic regulations (Fig. 3) and had a high degree of homogeneity for external, identified, and intrinsic regulations (Table 1). The smallest class, high combined (*n* = 25, 14.8%), was characterized by high scores on all exercise regulations (Fig. 3) and had a high degree of homogeneity for all exercise regulations (Table 1). Entropy for the three-class model was high (.89), and average classification probabilities were as follows: .96 (moderate combined), .96 (high autonomous), and .94 (high combined).

### Motivational profiles and baseline factors

There were no differences across motivational profile in age, race, ethnicity, baseline BMI, waist circumference, or MVPA (Table 2). Across the total sample, men were more likely to be classified to the moderate combined group (*P* = 0.048).

### Motivational profiles and physical activity

**Fig. 4:**
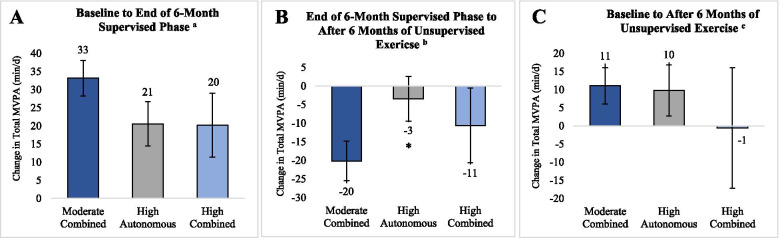
**A-C** Change in Mean Total MVPA over time across Baseline Motivational Profiles. Mean difference ( ± SE) in change in total MVPA (min/d) across profiles tested with Wald test and subsequent between group comparisons; * indicates significant difference (*P* < 0.05) from moderate combined profile; MVPA: minutes of moderate-to-vigorous physical activity. ^a^ For Panel A sample sizes are as follows: *n* = 64 for Moderate Combined; *n* = 36 for High Autonomous; *n* = 13 for High Combined. ^b^ For Panel B sample sizes are as follows: *n* = 57 for Moderate Combined; *n* = 30 for High Autonomous; *n* = 11 for High Combined. ^c^ For Panel C sample sizes are as follows: *n* = 58 for Moderate Combined; *n* = 30 for High Autonomous; *n* = 12 for High Combined

Motivational profiles were not different in terms of changes in MVPA from baseline to the end of the supervised exercise phase (Fig. 4A), or from baseline to the end of the unsupervised phase (Fig. 4C). However, during the transition from supervised to unsupervised exercise, there were differences across motivational profile in change in MVPA; on average, MVPA decreased within all three classes, but individuals in the high autonomous profile demonstrated the least decrease in MVPA compared to individuals in the moderate combined profile (Fig. 4B). After conducting a sensitivity analysis that assumed baseline values for missing follow-up MVPA values, this association was no longer significant (see Additional file 2, Supplementary Table S2). Study drop-out rate 12 months after starting the supervised exercise intervention was different across motivational profile (23% in Moderate Combined; 21% in High Autonomous; and 48% in High Combined; χ^2^ = 7.40, *P* = 0.02). Randomized group did not significantly influence the association between motivational profile and changes in MVPA. In addition, age, race, ethnicity, and baseline BMI and waist circumference were tested as potential covariates and/or as moderators and no variable was associated with either motivational profiles or changes in MVPA.

### Exploratory analyses of sex as a moderator

Given that sex was associated with motivational profile, and that sex was associated with change in MVPA from baseline to the end of the 6-month supervised exercise phase (women: 24 ± 4, men: 45 ± 8 min/d; *P* = 0.03), exploratory analyses tested sex as a moderator of class membership on change in MVPA from baseline to the end of the 6-month supervised exercise phase. There was a significant interaction between sex and motivational profile for the association between motivational profile and change in MVPA (Additional file 3). Men in the moderate combined group demonstrated a significantly greater increase in MVPA from baseline compared to women in each of the three motivational profiles. It is difficult to interpret sex differences within other motivational profiles given the small sample of men (*n* ≤ 3 per motivational profile, Additional File 3).

## Discussion

The goals of this study were to a) examine baseline motivational profiles for exercise utilizing a latent profile analysis approach in adults with overweight or obesity, and b) determine the association between motivational profiles and change in device-measured MVPA in the context of a comprehensive behavioral weight loss program. Three baseline motivational profiles emerged: high autonomous, high combined, and moderate combined. Results suggest that there were no differences across motivational profile in baseline level of device-measured MVPA or change in MVPA over 6 months of supervised exercise. However, adults motivated by autonomous reasons (value benefits of exercise, enjoyment) were more likely to sustain increases in MVPA during subsequent unsupervised exercise, once exercise support was removed. These results may suggest that adults with moderate-to-high scores on all types of exercise regulations may need additional long-term support in order to sustain their initial increases in MVPA.

The high autonomous profile was characterized by individuals who engage in exercise to achieve a valued outcome (identified exercise regulation) and because they enjoy the activity (intrinsic exercise regulation) and not for external recognition or reward. The high combined profile was characterized by individuals who engage in exercise for a variety of motives including: for social recognition or reward (external regulation), as a means of relieving guilt (introjected regulation), an inherent pleasure for PA (intrinsic regulation), and as a means of achieving valued outcomes such as health (identified regulation). Lastly, the moderate combined profile was characterized by individuals engaging in exercise for a variety of reasons, with moderate scores on all exercise regulations. This profile solution has similarities to previous studies that have identified clusters based on regulations for exercise [19–23].

There were no differences across motivational profile in baseline level of device-measured MVPA. These results are not consistent with previous cross-sectional studies. In a sample of 2473 adults, Friederichs et al. (2015) found that adults with an autonomous profile engaged in significantly higher levels of self-reported MVPA compared to those with controlled or low motivation profiles. In a sample of 351 adults with Type 2 Diabetes, Gourlan et al. (2016) found that adults with both the high combined and the self-determined profiles engaged in significantly higher self-reported PA compared to adults with the moderate profile. Although these studies included larger samples as compared to the present study, they relied on self-reported PA, which is prone to error and bias. It is possible that the use of self-reported PA may have biased the association between motivational profile and PA in a socially desirable way, such that participants aligned their self-reported levels of PA with their response to the motivation for exercise questions. Future studies should explore the association between motivational profile and device-measured PA in a larger sample.

Change in MVPA during the transition from supervised to unsupervised exercise was significantly different across motivational profile. All motivational profiles demonstrated a decrease in MVPA during the transition from supervised to unsupervised exercise; however, the high autonomous profile demonstrated the least amount of attenuation in MVPA as compared to the moderate combined profile. This finding is novel, given that no other study has reported on the association between motivational profile membership and longitudinal changes in PA. Generally, once support is lessened or removed in a weight loss intervention, participants demonstrate a decline in their adherence to PA [44]. Furthermore, a recent meta-analysis by McEwan et al. suggests that while PA significantly increased from baseline to post-intervention, PA significantly decreased from post-intervention to follow-up, demonstrating that improvements in PA are not sustained long-term [45]. Our results are consistent with these previous studies [44, 45]. It is possible that the combination of high levels of autonomous motives with low levels of external regulation and moderate introjected regulation is protective for adhering to PA once support is removed. Perhaps the 6-month supervised exercise program reinforced external regulations for exercise and thus, negatively impacted maintenance of PA long-term for participants with lower levels of autonomous motives, as indicated by the sharp decrease in MVPA levels in the moderate combined profile. This relationship is well-aligned with the SDT, which states that behaviors which are controlled by external factors (rewards, punishment, or self-imposed pressures) are predicted to only last as long as those contingencies or pressures remain in place [11].

Therefore, in order to promote the levels of PA recommended for weight management in current PA guidelines, weight loss interventions might benefit from focusing less on structured exercise supervision and more on increasing individuals’ identified and intrinsic regulations for exercise at the initiation of treatment (i.e. help participants find types of PA they enjoy, find value in PA). Recently, Teixeira and colleagues [46] published a classification of motivation and behavior change techniques that could be used to foster improvements in autonomous motivation in the context of a supervised exercise intervention. Examples include 1) prompting identification of important life aspirations/values/interests and exploring how changes in PA could be linked to them, 2) expressing positive support, regardless of success or failure, and 3) providing information to manage and limit effects of pressuring contingences that would undermine competence (such as extrinsic rewards, criticism, or negative feedback), among others [46]. These techniques could be accomplished through the provision of individualized support sessions or group-based classes with a staff member. Alternatively, participants with moderate-to-high scores on external regulations may need additional long-term support (e.g., continued supervised exercise, provision of an activity tracker, text message prompts/reminders, or periodic coaching calls) in order to sustain their initial increases in PA. Future studies should continue to explore the associations between motivational profiles and longitudinal changes in PA over time to further elucidate the combinations of exercise regulations that influence long-term adherence to PA.

After conducting a sensitivity analysis, imputing missing PA values, the results were no longer statistically significant. However, the pattern of response was similar (participants in the high autonomous profile demonstrated the least attenuation in MVPA during the transition from supervised to unsupervised exercise). Interestingly, motivational profile was associated with study dropout rate. Participants in the high combined profile were more likely to drop out of the study as compared to participants in either the moderate combined or the high autonomous profiles. Thus, the association between motivational profile and changes in MVPA once supervision was removed may be attenuated due to drop out rate. It is possible that the moderate-to-high levels of external and introjected regulations in the high combined profile were detrimental to remaining in the study to complete 12 months of an exercise intervention. Strong controlled exercise regulations might indicate a need for additional support for long-term engagement with the study protocol. This is the first study to report on the association between motivational profile and dropout rate. Future studies should continue to explore whether there is a link between motivation for exercise and likelihood of dropping out from a behavioral weight loss intervention.

Men in the moderate combined profile demonstrated a significantly greater increase in MVPA from baseline to the end of the 6-month supervised exercise phase, as compared to women within the same profile. Of the 28 men included in this analysis, the majority (75%) were classified in the moderate combined profile and these male participants seemed to drive the average increases in MVPA observed in this profile over the first 6 months. It is possible that men with moderate levels of motives on all exercise regulations responded the best to the supervised exercise intervention. Conversely, it may be that this motivational profile is more common among men and men in general respond better to a supervised exercise program. However, the exploratory sex results in our study should be interpreted with caution given the small number of men included in the analyses and that all participants self-selected in terms of volunteering for a weight loss study. Future studies with a larger sample and more men should be conducted to examine the role of sex on the association between motivational profile and longitudinal changes in MVPA.

Our sample was predominantly female, non-Hispanic White, and motivated to enroll in a comprehensive behavioral weight loss program which included a supervised exercise program. Amotivation for exercise was not included as part of the motivational profile make-up, since 66% of participants self-scored 0 for amotivation for exercise, as expected for individuals motivated to start a weight loss study that included exercise. Therefore, our results may not generalize to other demographic groups or to less motivated individuals. Our relatively small sample may have a) allowed for under extraction of latent profiles, relative to a larger sample size, and b) limited our ability to examine between-profile differences for change in MVPA outcomes. Although no theoretical formula is available for predicting power in a latent profile analysis, sample sizes found in previous studies [21, 47] suggest that 169 participants is sufficient to determine meaningful subgroups within the population. Despite this limitation, three profiles emerged, indicating that motivational heterogeneity was observed. Motivation for exercise was only captured at baseline; thus, this analysis did not ascertain whether motivation changed over time. If motivation and class membership are dynamic, future studies should consider adopting a complex adaptive systems approach to understand dynamic changes in motivation and MVPA over time and develop interventions tailored to real-life situations and changing contexts [48, 49]. Lastly, this was a secondary data analysis using a longitudinal, non-experimental design, thus it is difficult to draw conclusions about causality. However, the longitudinal study design allowed us to examine between-profile differences in changes in device-measured MVPA over time, filling an important gap in current literature. The use of a device to measure MVPA was a study strength, as device-based measures are more reliable and valid compared to self-reported methods of assessing PA [50].

## Conclusion

This study provides new insight into how a) different types of motivation for exercise coexist within an individual, and b) motivational profiles differ with respect to device-measured MVPA at baseline and changes in MVPA in adults with overweight or obesity enrolled in a behavioral weight loss intervention involving supervised exercise. Motivational profiles did not differ in baseline level of MVPA or change in MVPA over the 6-month supervised exercise intervention. However, once exercise supervision and support was removed, adults in the high autonomous motivational profile were protected against the standard attenuation in MVPA following removal of support/supervision. Future studies should examine changes in motivational profile over time and relationship to changes in device-measured PA as well as evaluate the effect of interventions targeted to increases autonomous motivation in those without high levels of these regulations at baseline. Results may allow us to provide a more targeted approach for treating adults with overweight or obesity.

## Supplementary Information

**Additional file 1 **: **Supplementary Table S1**. Fit Statistics for Latent Profile Analysis. Fit statistics from class enumeration process using latent profile analysis; Two to seven latent profile solutions were estimated during the class enumeration process to determine the optimal number of classes. Consistent with recent latent profile analysis methodological work [40], four different formulations for the covariance structure were tested.

**Additional file 2 **: **Supplementary Table S2**. Association between Motivational Profile and PA Outcomes. Mean difference in PA outcomes across profiles tested with Wald test and subsequent between group comparisons.

**Additional file 3 **: **Supplementary Figure S1**. Exploratory Results of Sex as a Moderator. Predicted change in mean ± standard error for total MVPA (min/d) levels from baseline to the end of the 6-month supervised exercise program by sex and motivational profile.

## Data Availability

Deidentified individual participant data that underlie the results reported in this article will be made available for 5 years following article publication to researchers who provide methodologically sound proposals. To gain access, data requestors will need to sign a data sharing agreement. Proposals for deidentified data and/or a full trial protocol should be directed to vicki.catenacci@cuanschutz.edu. For readers interested in obtaining access to the analytical code and output, requesters can email the first author at danielle.ostendorf@cuanschutz.edu.

## Abbreviations

BMI: Body Mass Index
BREQ-2: Behavioral Regulation in Exercise Questionnaire
CU-AHWC: University of Colorado Anschutz Health and Wellness Center
MVPA: Moderate-to-Vigorous Physical Activity
PA: Physical Activity
SDT: Self-Determination Theory
