# Supplementary material for: Motivational profiles and change in physical activity during a weight loss intervention: a secondary data analysis
Source: Int J Behav Nutr Phys Act. 2021 Dec 4;18:158. doi: 10.1186/s12966-021-01225-5 (PMC8642857; doi:10.1186/s12966-021-01225-5)
Supplement: Supplementary file 1 — Additional file 1 : Supplementary Table S1. Fit Statistics for Latent Profile Analysis. Fit statistics from class enumeration process using latent profile analysis; Two to seven latent profile solutions were estimated during the class enumeration process to determine the optimal number of classes. Consistent with recent latent profile analysis methodological work [40], four different formulations for the covariance structure were tested. [file 12966_2021_1225_MOESM1_ESM.docx]

**Additional File 1**

**Supplementary Table S1:** Fit Statistics for Latent Profile Analysis, Steps 1-4

|  |  |  |  |  |  |  | **H0: K classes; H1: K + 1 classes** | | | | |
| --- | --- | --- | --- | --- | --- | --- | --- | --- | --- | --- | --- |
| Model (K-class) | **LL** | **npar** | **AIC** | **BIC** | **CAIC** | **AWE** | **LRT** | **Adj LMR p-value** | **BLRT**  **p-value** | **BF**  **(K, K+1)** | **cmP (K)** |
| **Step 1** |  |  |  |  |  |  |  |  |  |  |  |
| 1-class | -937.05 | 8 | 1890.12 | 1915.16 | 1923.16 | 1980.20 |  |  |  | 0.00 | 2E-29 |
| 2-class | -874.92 | 13 | 1775.83 | 1816.52 | 1829.52 | **1922.21** | 119.63 | **<0.01** | **<0.01** | 0.00 | 5E-08 |
| 3-class | -858.72 | 18 | 1753.43 | 1809.77 | 1827.77 | 1956.11 | 31.18 | 0.18 | **<0.01** | 0.00 | 1E-06 |
| 4-class | -832.92 | 23 | 1711.84 | **1783.83** | **1806.83** | 1970.82 | 26.24 | **0.04** | **<0.01** | **181.32** | **0.62** |
| 5-class | -820.70 | 28 | 1697.40 | 1785.04 | 1813.04 | 2012.68 | 23.52 | 0.39 | **<0.01** | 99.14 | 0.34 |
| 6-class | -810.03 | 33 | 1686.06 | 1789.34 | 1822.34 | 2057.63 | 20.54 | 0.23 | **<0.01** | 11.52 | 0.04 |
| 7-class | -799.65 | 38 | 1675.30 | 1794.23 | 1832.23 | 2103.17 | 19.98 | 0.54 | **<0.01** | 1.00 | 3E-03 |
| **Step 2** |  |  |  |  |  |  |  |  |  |  |  |
| 1-class | -937.06 | 8 | 1890.12 | 1915.16 | 1923.16 | 1980.20 | - | - | - | 0.00 | 0.00 |
| 2-class | -868.06 | 17 | 1770.12 | 1823.33 | 1840.33 | **1961.54** | 135.08 | **<0.01** | **<0.01** | 0.83 | 0.00 |
| 3-class | -835.14 | 26 | 1722.28 | **1803.66** | **1829.66** | 2015.03 | 64.45 | **0.02** | **<0.01** | **15476.66** | **0.71** |
| 4-class | -812.95 | 35 | 1695.90 | 1805.44 | 1840.44 | 2089.99 | 43.44 | 0.28 | **0.04** | 6326.76 | 0.29 |
| 5-class | -798.62 | 44 | 1685.95 | 1822.95 | 1866.95 | 2180.67 | 28.06 | 0.74 | 0.38 | 1.00 | 0.00 |
| **Step 3** |  |  |  |  |  |  |  |  |  |  |  |
| 1-class | -850.62 | 14 | 1729.24 | 1773.06 | 1787.06 | 1886.88 |  |  |  | 0.00 | 4E-11 |
| 2-class | -816.99 | 19 | 1671.98 | 1731.45 | 1750.45 | **1885.92** | 64.74 | **<0.01** | **<0.01** | 7136.57 | 4E-02 |
| 3-class | -801.28 | 24 | 1650.56 | **1725.68** | **1749.68** | 1920.80 | 30.24 | 0.10 | **<0.01** | **127674.63** | 7E-01 |
| 4-class | -789.73 | 29 | 1637.46 | 1728.23 | 1757.23 | 1963.99 | 22.24 | **0.048** | **<0.01** | 35720.98 | **0.21** |
| 5-class | -782.73 | 34 | 1633.47 | 1739.88 | 1773.88 | 2016.30 | 13.47 | 0.59 | 0.24 | 105.19 | 0.00 |
| 6-class | -774.56 | 39 | 1627.13 | 1749.19 | 1788.19 | 2066.26 | 15.72 | 0.34 | 0.07 | 1.00 | 0.00 |
| **Step 4** |  |  |  |  |  |  |  |  |  |  |  |
| 1-class | -850.62 | 14 | 1729.24 | 1773.06 | 1787.06 | **1886.88** | - | - | - | 0.00 | 0.00 |
| 2-class | -795.36 | 29 | 1648.73 | **1739.49** | **1768.49** | 1975.26 | 109.10 | **<0.01** | **<0.01** | **33098.40** | **1.00** |
| 3-class | -767.30 | 44 | 1622.59 | 1760.31 | 1804.31 | 2118.02 | 55.41 | 0.08 | 0.06 | 1.00 | 0.00 |
| 4-class | Not well-identified | | | | | |  |  |  |  |  |

**Legend for Supplementary Table S1:** Fit statistics from class enumeration process using latent profile analysis; Two to seven latent profile solutions were estimated during the class enumeration process to determine the optimal number of classes. Consistent with recent latent profile analysis methodological work (1), four different formulations for the covariance structure were tested. Step 1: covariances fixed to zero and variances are the same across classes, Step 2: covariances are fixed to zero, but variances are allowed to differ across classes, Step 3: covariances are allowed to differ across classes, but variances are the same across classes, and Step 4: covariances and variances were allowed to differ across classes; Although most prior applications of latent profile analysis have relied exclusively on the variance/covariance structure estimated under the most strict covariance structure, recent evidence has recommended empirical tests of the conditional independence assumption (that the latent constructs accounts for all covariation among indicators) as performed in less strict covariance structures (1); LL: Model Log-likelihood; npar: number of parameters; AIC: Akaike Information Criteria; BIC: Bayesian Information Criteria; CAIC: Consistent Akaike’s Information Criteria; AWE: Approximate Weight of Evidence Criterion; BLRT: Bootstrap Likelihood Ratio Test; Adj LMR: Adjusted Lo-Mendell-Rubin Likelihood Ratio Test; BF: Bayes Factor; cmP: Correct Model Probability; Bolded values correspond to “best” model according to the fit index – columns without bolded values indicate the best value for that index was not reached prior to the maximum class extraction supported by the data.

**References**

1. Meyer JP, Morin AJS. A person-centered approach to commitment research: Theory, research, and methodology. J Organ Behav. 2016;37(4):584-612.
