# Supplementary material for: Motivational profiles and change in physical activity during a weight loss intervention: a secondary data analysis
Source: Int J Behav Nutr Phys Act. 2021 Dec 4;18:158. doi: 10.1186/s12966-021-01225-5 (PMC8642857; doi:10.1186/s12966-021-01225-5)
Supplement: Supplementary file 2 — Additional file 2 : Supplementary Table S2. Association between Motivational Profile and PA Outcomes. Mean difference in PA outcomes across profiles tested with Wald test and subsequent between group comparisons. [file 12966_2021_1225_MOESM2_ESM.docx]

**Additional File 2**

**Supplementary Table S2:** Association between Motivational Profile and PA Outcomes

| **PA Outcomes (min/d)** | **Moderate Combined (MC)** | | **High Autonomous (HA)** | | **High Combined (HC)** | | **Overall**  ***P* value** | **MC:HA**  **P value** | **MC:HC**  ***P* value** | **HA:HC**  ***P* value** |
| --- | --- | --- | --- | --- | --- | --- | --- | --- | --- | --- |
|  | *n* | Mean ± SE | *n* | Mean ± SE | *n* | Mean ± SE |  |  |  |  |
| ***Change in PA (End of 6-Month Supervised Phase – Baseline)*** | | | | | | | | | | |
| *Total MVPA* | 64 | 33 ± 5 | 36 | 21 ± 6 | 13 | 20 ± 9 | 0.22 | 0.12 | 0.21 | 0.98 |
| *Total MVPA, Imputed ^a^* | 84 | 25 ± 4 | 48 | 15 ± 5 | 22 | 12 ± 6 | 0.12 | 0.13 | 0.05 | 0.62 |
| ***Change in PA (End of 6-Month Unsupervised Phase – End of 6-Month Supervised Phase)*** | | | | | | | | | | |
| *Total MVPA* | 57 | -20 ± 5 | 30 | -3 ± 6 | 11 | -11 ± 10 | 0.13 | **0.04** | 0.41 | 0.54 |
| *Total MVPA, Imputed ^a^* | 86 | -17 ± 4 | 50 | -8 ± 5 | 22 | -12 ± 7 | 0.41 | 0.18 | 0.58 | 0.66 |
| ***Change in PA (End of 6-Month Unsupervised Phase - Baseline)*** | | | | | | | | | | |
| *Total MVPA* | 58 | 11 ± 5 | 30 | 10 ± 7 | 12 | -1 ± 17 | 0.80 | 0.88 | 0.51 | 0.57 |
| *Total MVPA, Imputed ^a^* | 84 | 8 ± 4 | 48 | 6 ± 4 | 22 | 0 ± 9 | 0.71 | 0.78 | 0.41 | 0.52 |

**Legend for Supplementary Table S2:** Mean difference in PA outcomes across profiles tested with Wald test and subsequent between group comparisons; Statistically significant *P* values (*P* < 0.05) are indicated in bold; MVPA: minutes of moderate-to-vigorous physical activity; PA: physical activity.

^a^ Missing values imputed with baseline observation carried forward.
