# Supplementary material for: Motivational profiles and change in physical activity during a weight loss intervention: a secondary data analysis
Source: Int J Behav Nutr Phys Act. 2021 Dec 4;18:158. doi: 10.1186/s12966-021-01225-5 (PMC8642857; doi:10.1186/s12966-021-01225-5)
Supplement: Supplementary file 3 — Additional file 3 : Supplementary Figure S1. Exploratory Results of Sex as a Moderator. Predicted change in mean ± standard error for total MVPA (min/d) levels from baseline to the end of the 6-month supervised exercise program by sex and motivational profile. [file 12966_2021_1225_MOESM3_ESM.docx]

**Additional File 3**

**Supplementary Figure S1:** Exploratory Results of Sex as a Moderator

Sex *P* = 0.58

Motivational Profile *P* = **0.01**

Sex*Motivational Profile *P* = **0.01**

**Legend for Supplementary Figure S1:** Predicted change in mean ± standard error for total MVPA (min/d) levels from baseline to the end of the 6-month supervised exercise program by sex and motivational profile; * indicates significant difference from “moderate combined” profile within each gender; ^†^ indicates significant difference from men in the “moderate combined” profile. Results from linear regression; MVPA: minutes of moderate-to-vigorous physical activity; Sample sizes are as follows: *n* = 50 for women and n = 14 for men in Moderate Combined; *n* = 33 for women and n = 3 for men in High Autonomous; *n* = 11 for women and n = 2 for men in High Combined.
